# Chemical Proteomic Approach for In-Depth Glycosylation Profiling of Plasma Carcinoembryonic Antigen in Cancer Patients

**DOI:** 10.1016/j.mcpro.2023.100662

**Published:** 2023-10-10

**Authors:** Jin Chen, Lijun Yang, Chang Li, Luobin Zhang, Weina Gao, Ruilian Xu, Ruijun Tian

**Affiliations:** 1 Department of Chemistry and Research Center for Chemical Biology and Omics Analysis, School of Science, Southern University of Science and Technology, Shenzhen, China; 2 Clinical Center for Molecular Diagnosis and Therapy, The Second Affiliated Hospital of Fujian Medical University, Quanzhou, Fujian, China; 3 Department of Oncology, The Second Clinical Medical College, Jinan University (Shenzhen People's Hospital), Shenzhen, China; 4 The First Affiliated Hospital, Jinan University, Guangzhou, China

**Keywords:** carcinoembryonic antigen, plasma, glycosylation, mass spectrometry, site-specific glycoforms

## Abstract

Carcinoembryonic antigen (CEA) of human plasma is a biomarker of many cancer diseases, and its N-glycosylation accounts for 60% of molecular mass. It is highly desirable to characterize its glycoforms for providing additional dimension of features to increase its performance in prognosis and diagnosis of cancers. However, to systematically characterize its site-specific glycosylation is challenging because of its low abundance. Here, we developed a highly sensitive strategy for in-depth glycosylation profiling of plasma CEA through chemical proteomics combined with multienzymatic digestion. A trifunctional probe was utilized to generate covalent bond of plasma CEA and its antibody upon UV irradiation. As low as 1 ng/ml CEA in plasma could be captured and digested with trypsin and chymotrypsin for intact glycopeptide characterization. Twenty six of 28 potential N-glycosylation sites were well identified, which were the most comprehensive N-glycosylation site characterization of CEA on intact glycopeptide level as far as we known. Importantly, this strategy was applied to the glycosylation analysis of plasma CEA in cancer patients. Differential site-specific glycoforms of plasma CEA were observed in patients with colorectal cancers (CRCs) and lung cancer. The distributions of site-specific glycoforms were different as the progression of CRC, and most site-specific glycoforms were overexpressed in stage II of CRC. Overall, we established a highly sensitive chemical proteomic method to profile site-specific glycosylation of plasma CEA, which should generally applicable to other well-established cancer glycoprotein biomarkers for improving their cancer diagnosis and monitoring performance.

## Highlights

• A chemical proteomic approach for glycosylation profiling of low abundant proteins.
• In-depth profiling of plasma carcinoembryonic antigen on intact glycopeptide level.
• Glycosylation identification with high sensitivity and reproducibility.
• Glycosylation features of plasma carcinoembryonic antigen in cancer patients.

## Graphical Abstract

**Figure:**
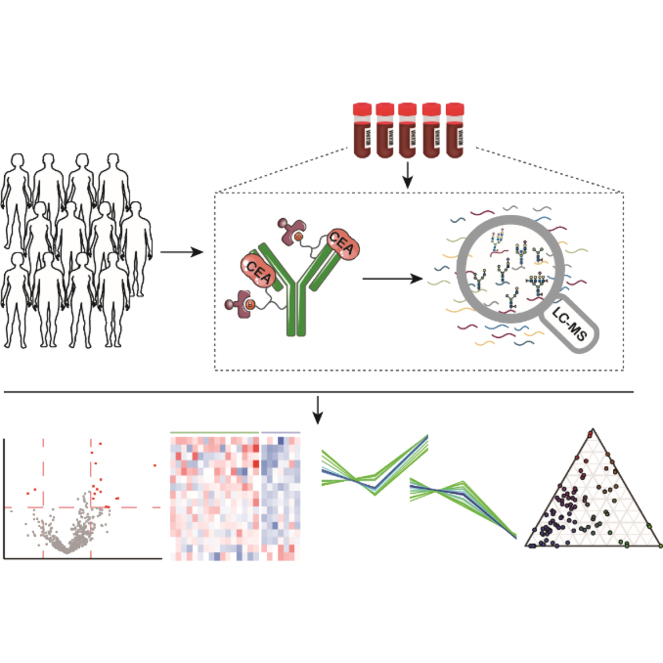

## In Brief

A chemical proteomic approach for glycosylation profiling of proteins was established for glycosylation characterization of plasma CEA with low abundance. Although CEA has been widely used in diagnosis and prognosis of many cancers, it lacks specificity and sensitivity. We found that the glycosylation of CEA on intact glycopeptide level provided additional dimension of molecular features to improve the performance of CEA in cancer diagnosis and progression.

Protein glycosylation is a crucial post-translational modification in health and disease (1). Evidences showed that glycosites and glycan structures are both essential for glycosylation and various biological processes. For example, N-glycosylation regulates cancer metastasis and invasion (2, 3), and the alternation of N-glycan was correlated with the development of cancer (4). PD-L1 contributes to the immune escape of cancer cell, and its N-linked glycosylation at sites N35, N192, N200, and N219 is crucial for its stability and binding to PD-1 (5, 6). Proteins with glycosylation, such as carbohydrate antigens, has been used as cancer biomarkers for decades (7). However, their roles in cancer diagnosis and prognosis are with limited sensitivity and specificity (8). It is highly desirable to provide other molecular features, such as the glycosylation sites and glycans, to increase their performance in cancer diagnosis.

Plasma is the predominant source for clinical diagnostic analyses as its collection is noninvasive, and most Food and Drug Administration–approved plasma biomarkers are with low concentration (9, 10). Functional biomarkers often secrete from tissues into circulation system, but it is a challenge to identify them because of their low abundance and the complexity of plasma (11, 12). Antibody is widely used to capture target proteins and applied to immunoprecipitation and ELISA detection, but their affinity is often limited, especially with the presence of complex plasma components (13, 14). Chemical proteomics is a powerful tool for covalently labeling targeted proteins in complex biological systems followed with enrichment and mass spectrometry (MS) analysis (15). For example, an active-site directed probe could be used to profile reactive residues of proteins with high sensitivity (16). We have synthesized a trifunctional probe that could selectively recognize tyrosine phosphorylation (pTyr) and efficiently covalently crosslink pTyr-dependent protein complexes in complex clinical samples (17, 18). It is therefore desirable to adopt chemical proteomic approach for profiling targeted proteins and their modifications in plasma samples.

Carcinoembryonic antigen (CEA) serves as a popular biomarker in the diagnosis and prognosis of many cancers such as colorectal cancer (CRC) and lung cancer, and its level of >5 ng/ml was considered abnormal (19, 20). However, the overexpression of CEA could not often be observed in patients with cancer recurrence and could not apply for early diagnosis of cancer (21). CEA is a highly N-glycosylated protein, which accounts for 60% of molecular mass, and should provide additional dimensions of molecular characteristics for cancer diagnosis and prognosis. Lectin microarray containing 56 plant lectins was used to detect CEA glycosylation in CRC patients, which indicated upregulation of mannose, *N*-acetylgalactosamine, *N*-acetylglucosamine, and galactose at stage II of CRC (22). Alternatively, MALDI-TOF-MS has been used to profile 61 unique glycan patterns of purified CEA, which were released by PNGase F (23). To profile the glycosylated sites and glycans simultaneously, intact glycopeptides from 10 μg CEA purified from human CRC were analyzed by capillary electrophoresis–MS system (24). Among 28 potential N-linked glycosylation sites of CEA, 21 of them were successfully identified with the combination of multiple enzymes including trypsin, Glu-C, endoproteinase, and pronase (24). Collectively, it is desirable to set up a strategy with high sensitivity for exploring the additional dimension of CEA glycosylation features from clinical samples.

**Fig. 1:**
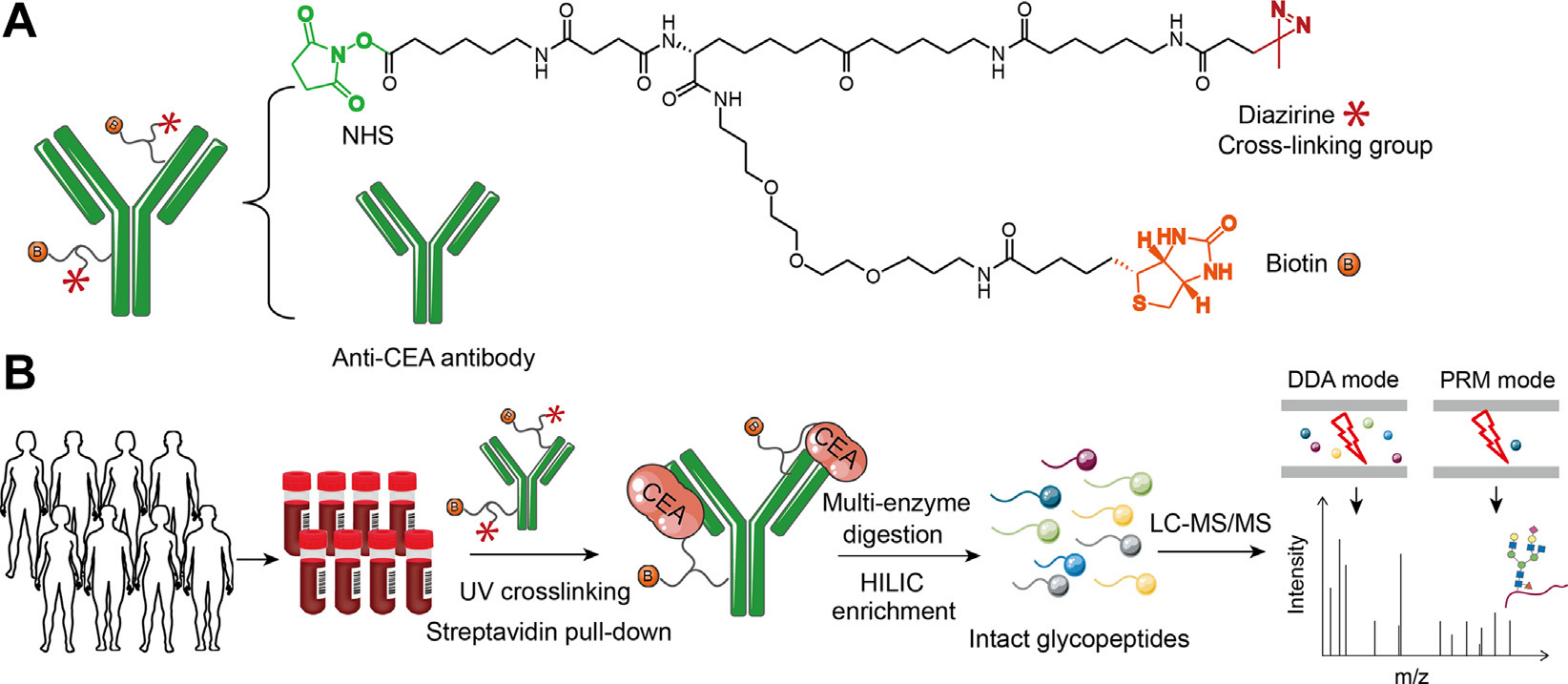
**The workflow of this study.***A*, the structure of the trifunctional probe that contains NHS, biotin, and diazirine groups. *B*, plasma was collected and incubated with labeled anti-CEA for capturing plasma CEA, followed with UV crosslinking and multienzymatic digestion. Intact glycopeptides were enriched by HILIC and then identified by LC–MS with DDA and PRM modes. CEA, carcinoembryonic antigen; DDA, data-dependent acquisition; HILIC, hydrophilic interaction chromatography; PRM, parallel reaction monitoring.

In this study, a chemical proteomic approach was established for in-depth glycosylation profiling of plasma CEA with high sensitivity. Taking advantage of a trifunctional probe, anti-CEA was first assembled with the probe that allowed to selectively capture, crosslink, and enrich CEA in plasma sample. The intact glycopeptides were released by multienzymatic digestion and analyzed by MS (Fig. 1). As much as 26 of 28 N-glycosylation sites were identified in plasma with 500 ng/ml CEA spiked in, and site-specific glycosylation of CEA could be profiled in plasma samples of individual cancer patients with concentration as low as 1 ng/ml. The unique features of site-specific glycoforms of plasma CEA in CRC and lung cancer were successfully characterized. Moreover, its distribution was different in CRC patients at different stages. Therefore, site-specific glycosylation of plasma CEA has the ability to describe the status of disease and provides potential for cancer diagnosis.

## Experimental Procedures

### Clinical Sample

Plasma samples were obtained from the Department of Oncology, Shenzhen People’s Hospital. The study was approved by the Institutional Ethical Review Boards of Shenzhen People’s Hospital (number: LL-KY-2021169) and performed according to the Declaration of Helsinki. Plasma samples were collected in EDTA tube at first morning during hospitalization. They were centrifugated within 6 h, and the upper were divided into two or three copies. They were then stored at −80 °C for further use. Detail information of clinical samples is listed in Supplemental Table S1.

### Sample Preparation

Anti-CEA antibody (catalog no.: 2383; Cell Signaling Technology) was labeled with the trifunctional probe based on our previous reports with minor modification (17). Briefly, 10 μl antibody was incubated with 1 μl 100 mM probe for 2 min at room temperature, and the reaction was stopped by glycine. The labeled anti-CEA was desalted by columns (Zeba spin desalting columns, 7 K molecular weight cutoff, catalog no.: 89882; Thermo Scientific) and incubated with 1 ml plasma sample with twofold dilution and 15 μl Sepharose streptavidin beads (catalog no.: 17511301; Cytiva) overnight. After brief centrifugation, the beads were transferred to 24-well plates for UV irradiation at 365 nm for 10 min at 4 °C. Next, the beads were washed with washing buffer 1 (radioimmunoprecipitation assay, catalog no.: P0013B; Beyotime) three times, followed with washing buffer 2 (50 mM NH_4_HCO_3_) two times. The captured proteins were alkylated and digested with trypsin as our previous reports (25). For multienzymatic digestion, chymotrypsin (catalog no.: V1061; Promega) or elastase (catalog no.: V1891; Promega) was further added according to the instruction of manufacturer. Finally, the peptides were desalted, and intact glycopeptides were enriched by ZIC-HILIC beads (Merck; product no.: 1.50458.0001) according to our previous study (25).

### LC–MS/MS Analysis

The intact glycopeptides were analysis by Q Exactive HF-X MS coupled with easy LC system (Thermo Scientific). The nanoLC separation was performed on a 15 cm × 100 μm i.d. C18 (1.9 μm, 120 Å) capillary column at a flow rate of 250 nl/min with a 60 min gradient (5% B to 25% B for 50 min, increase to 90% B at 51 min and hold for 9 min). Formic acid (0.1%, v/v) in water and in 80% acetonitrile (v/v) was used as mobile phase A and B, respectively. All MS spectra were acquired from *m*/*z* 400 to 1800 with a mass resolution of 120,000 in data-dependent acquisition (DDA) mode, in which the ten most intense ions were selected for MS/MS scan *via* stepped normalized collision energy (20%, 30%, and 40%). Tandem MS was acquired at a resolution of 15,000 and using an isolation window of 2.0 *m*/*z*.

Intact glycopeptides from clinical cohort were analyzed by Exploris 240 MS coupled with a Dionex UltiMate 3000 RSLCnano System (Thermo Scientific). The nanoLC separation was performed as following: 5 to 28% buffer B for 50 min, 28 to 55% buffer B for 20 min, 55 to 99% buffer B for 0.5 min, 99% buffer B for 9.5 min, and 99 and 4% buffer B for 0.5 min. MS parameters with DDA mode were the same as Q Exactive HF-X MS. In addition, parallel reaction monitoring (PRM) mode was utilized for detecting CEA intact glycopeptides, and the PRM list is shown in Supplemental Table S2.

### Data Processing

The identification of intact glycopeptides was performed by pGlyco3.0 (version 20210615) (26). The enzymes were set as follows: trypsin, C-term of KR with two missed cleavages; trypsin and chymotrypsin, C-term of KRFYLWM with six missed cleavages; and trypsin and elastase, C-term of KRLITSAV with six missed cleavages. Cysteine carbamidomethylation was set as fixed modification, and methionine oxidation and acetylation on protein N-term were set as variable modification. Precursor and fragment tolerance were respectively set as 10 and 20 ppm, and default false discovery rate (1% peptide and glycan) was set. CEA (UniProt; catalog no.: P06731) was set as database when standard CEA was used and data with PRM mode. Database downloaded from UniProt-human (in January 2019, 20,413 entries) was used when plasma samples were analyzed. Other parameters were set as default.

All the quantifications were processed by pGlycoQuant (version 2022.11), which could be used for quantitative glycoproteomics at intact glycopeptide level (27). Primary and tandem MS were used for intact glycopeptide quantitation. Raw mass spectrometric data and search results from pGlyco3.0 were input, and other parameters were set as default. Quantification of site-specific glycoforms could be acquired.

### Experimental Design and Statistical Rationale

In total, plasma from 42 patients were collected, and their detail information, such as sex, age, cancer type, and CEA level, are listed in Supplemental Table S1. Eight of them with CEA >500 ng/ml were used for investigating our chemical proteomic approach, and they were measured with three technical replicates. Then, 16 CRC patients and seven lung cancer patients with CEA >300 ng/ml were involved to investigate the CEA glycosylation in different cancers, which were identified by LC–MS with DDA mode. Three technical replicates were acquired, and the average intensities of CEA site-specific glycoforms were directly used because of their similar distribution. Site-specific glycoforms were excluded if they were not detected in more than 75% of the samples, and unpaired two-tailed Student’s *t* tests were utilized for differential analysis. Finally, 11 CRC patients with different stages, including three in stage II (CEA below 2 ng/ml), three in stage III (2 with CEA around 3 ng/ml and 1 with CEA 300 ng/ml), and five in stage IV (CEA above 250 ng/ml), were investigated. Their CEA glycosylations were detected by LC–MS with PRM mode. No technical replicates were acquired because of biological replicates. Quantified site-specific glycoforms were not further filtered. Volcano, heatmap, Pearson correlation, triangle graph, and cluster enrichment were performed using RStudio (RStudio, Inc) with packages, ggplot2, corrplot, ggtern, pheatmap, and mfuzz. SIMCA (version 14.1; Umetrics) was used for orthogonal partial least squares discriminant analysis (OPLS-DA) analysis.

## Results

### Development of the Chemical Proteomic Approach

We established a chemical proteomic approach for glycosylation of plasma CEA (Fig. 1). First, anti-CEA was covalently labeled by the trifunctional probe, which contains NHS, biotin, and diazirine groups (Fig. 1*A*). Free primary amines of N terminus or lysine of anti-CEA could be efficiently labeled *via N*-hydroxysuccinimide ester chemistry, which has been used for capturing pTyr signaling complexes in our previous work (17). Plasma sample of cancer patient was incubated with the labeled anti-CEA for capturing CEA (Fig. 1*B*). Then, covalent bonds between anti-CEA and CEA were efficiently generated *via* UV crosslinking group diazirine with ∼50 Å space arm (Fig. 1*B*). Low-abundant CEA could therefore be enriched from plasma samples by the biotin group. Taking advantage of covalent crosslinking and biotin–streptavidin recognition with high affinity, interferences from plasma sample with extremely high dynamic range could be efficiently removed by stringent washing.

To best explore the molecular signature of site-specific glycosylation, we identified potential glycosylation sites and their glycoforms through multienzymatic digestion and ZIC-HILIC-based intact glycopeptide enrichment. DDA coupled with liquid chromatography was first applied for identifying and quantifying the enriched intact glycopeptides, and PRM was further adopted for quantified identified intact glycopeptides with improved sensitivity (Fig. 1*B*). Collectively, we designed an integrated chemical proteomic pipeline for efficiently capturing and quantitatively analyzing site-specific glycoforms of CEA.

### Evaluation and Optimization of Chemical Proteomic Workflow

**Fig. 2:**
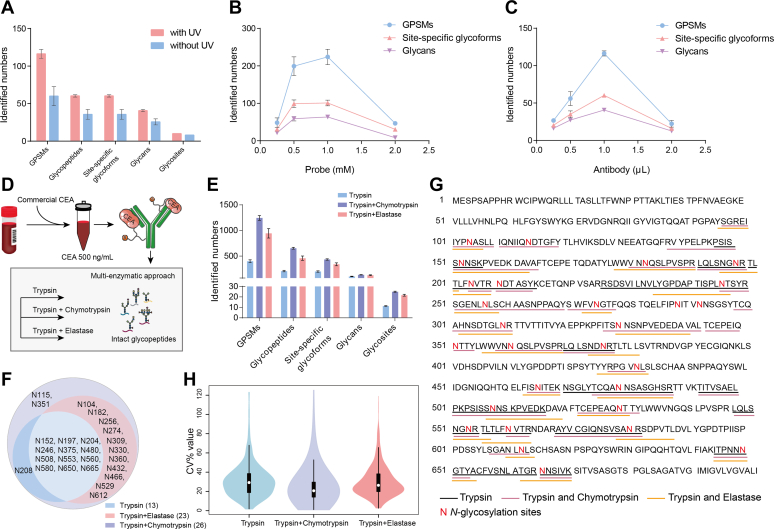
**Evaluation and optimization of the workflow for CEA glycosylation identification.** The number of glycopeptide-spectrum matches (GPSMs), glycopeptides, site-specific glycoforms, glycans, and glycosites was investigated with the condition of chemical labeling (with and without UV) (*A*), different concentrations of probe (*B*), and antibody (*C*). Probe (1 mM) and anti-CEA (1 μl) in the reaction with UV crosslinking showed best performance in CEA glycosylation identification. Commercial CEA protein was spiked in plasma with a final concentration of 500 ng/ml, followed with multienzymatic digestion (trypsin digestion, trypsin and elastase digestion, and trypsin and chymotrypsin digestion) for in-depth glycosylation identification (*D*). Much more GPSMs, glycopeptides, site-specific glycoforms, glycans, and glycosites could be identified in trypsin and chymotrypsin digestion (*E*). In comparison, 13, 23, and 26 CEA glycosylation sites were identified in trypsin digestion, trypsin and elastase digestion, and trypsin and chymotrypsin digestion, respectively (*F*). The identified glycosylated peptide sequences with multienzymatic approach were shown (*G*). Three digestion strategies have comparable quantification performance, which is acceptable for clinical cohort study (*H*). CEA, carcinoembryonic antigen.

First, we investigated the chemical labeling workflow with the readout of identified intact glycopeptides of CEA in plasma, and trypsin was used for protein digestion. The number of glycopeptide-spectrum matches (GPSMs) showed nearly twofold increase, and glycopeptides and site-specific glycoforms exhibited greatly increase with about 70% improvement with UV compared to without UV irradiation (Fig. 2*A*, Supplemental Table S3). Compared with without UV irradiation, much less peptides from nonspecific proteins were observed, and GPSMs of CEA accounted for a twofold higher proportion of all GPSMs with UV (Supplemental Fig. S1). This indicated less peptides from nonspecific proteins, and glycosylated peptide enrichment greatly improved CEA glycosylation identification. Glycans and glycosites were also greatly increased with UV irradiation. These results indicated that covalent linkage between anti-CEA and CEA greatly contributed to the improvement of glycosylation identification of plasma CEA.

Anti-CEA was labeled with probe *via N*-hydroxysuccinimide ester chemistry, and its free amino group would be replaced so that its binding affinity might be changed. We thus investigated the labeling reaction and optimized the concentration of probe and antibody for intact glycopeptide identification. The concentration of trifunctional probe greatly affected CEA glycosylation identification. Both 0.5 mM and 1 mM probe had better performance, whereas 1 mM probe performed the best for identifying slightly more in GPSMs and glycan patterns (Fig. 2*B*, Supplemental Table S4). The amount of anti-CEA also significantly affected intact glycopeptide identification, among which 1 μl anti-CEA has the best performance for GPSMs, site-specific glycoforms, and glycan pattern identification (Fig. 2*C*, Supplemental Table S5). Therefore, we used 1 mM probe and 1 μl anti-CEA in the reaction for better CEA glycosylation identification.

To increase the depth of CEA glycosylation identification, multienzymatic digestion approach was adopted by using trypsin and trypsin paired with either chymotrypsin or elastase. Twenty-six glycosites of commercial CEA were identified in trypsin and chymotrypsin digest as well as trypsin and elastase digest (Supplemental Table S6), which indicated the in-depth glycosylation coverage of CEA by multienzymatic digestion approach. Next, CEA was spiked in plasma with a final concentration of 500 ng/ml and was pulled down by the trifunctional probe-labeled anti-CEA followed with a tryptic digestion and two types of multienzymatic digestions (Fig. 2*D*). Compared with trypsin digestion and trypsin combined with elastase digestion, trypsin combined with chymotrypsin digestion showed much more identified GPSMs, glycopeptides, site-specific glycoforms, and glycosites (Fig. 2*E*, Supplemental Table S7). In comparison, 13, 23, and 26 CEA glycosylation sites were identified in trypsin digestion, trypsin and elastase digestion, and trypsin and chymotrypsin digestion, respectively (Fig. 2*F*), and their identified glycopeptide sequences were shown (Fig. 2*G*). The glycosylation of N288 and N292 could not be observed in our multienzymatic approach. Trypsin and chymotrypsin approach has achieved 26 glycosites, which showed the best performance in the glycosylation identification of CEA in plasma as far as we know. Importantly, three digestion strategies have comparable quantification performance, which is acceptable for clinical cohort study (Fig. 2*H*, Supplemental Table S8). Collectively, we adopted trypsin and chymotrypsin for digestion in our workflow for further clinical analysis.

### Profiling of Glycosylation of Plasma CEA in Clinical Samples

**Fig. 3:**
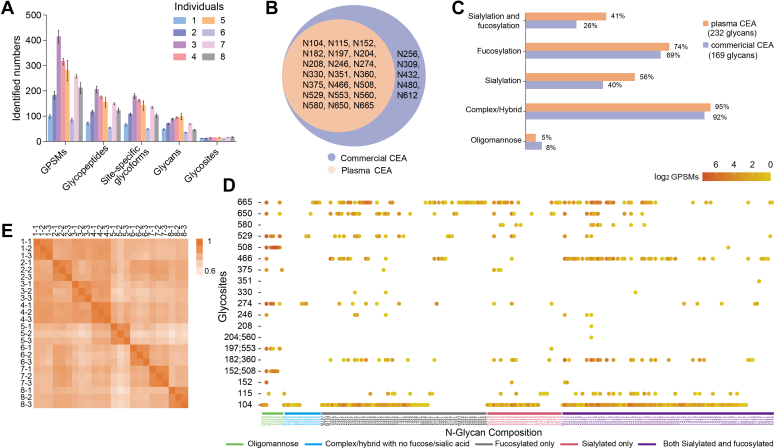
**Glycosylation analysis of plasma CEA from eight individuals.** GPSMs, glycopeptides, site-specific glycoforms, and glycosites of CEA in different clinical samples were shown in different clinical samples, which indicates their difference (*A*). Twenty-six and 21 glycosylation sites were identified in commercial CEA and plasma samples, respectively (*B*), and 232 and 169 glycans in plasma and commercial CEA were identified, respectively (*C*). Their identified intact glycopeptides were shown with detailed molecular features. Its *color* represented their GPSMs (*D*). The Pearson correlation coefficients of three replicates were high with a range of 0.91 to 0.97, which indicated high reproducibility (*E*). CEA, carcinoembryonic antigen; GPSM, glycopeptide-spectrum match.

Our workflow was then applied to eight individuals whose CEA were above 500 ng/ml and MS with DDA mode was utilized (Supplemental Table S1, Fig. 1*B*). Expectedly, we identified a large number of GPSMs, glycopeptides, site-specific glycoforms, and glycosites in different clinical samples, which indicated the differential glycosylation of plasma CEA between individual patients (Fig. 3*A*, Supplemental Table S9). Twenty-one CEA glycosylation sites were identified, whereas N256, N309, N432, N480, and N612 could rarely be observed compared with commercial CEA (Fig. 3*B*). The decrease of identified glycosites might be due to the differential CEA glycosylation in individuals. Interestingly, 232 and 169 glycans in plasma and commercial CEA were identified respectively, and their quantified CEA levels were roughly equal (Supplemental Fig. S2), demonstrating the much higher diversity of its glycosylation in real-world clinical samples. The plasma CEA and the commercial CEA protein did not show great difference in the distribution of modification with oligomannose, complex, or hybrid N-glycans. They also displayed nearly the same proportion of fucosylation, which accounted for around 70% of glycan patterns (Fig. 3*C*). Sialylated glycopeptides with fucosylation had nearly 60% increase in plasma CEA compared with commercial CEA, whereas those without fucosylation occupied similar percentage (Fig. 3*C*). It indicated that sialylation peptides of CEA were likely to be modified with fucose in patients with CEA overexpression.

The identified intact glycopeptides were shown with detailed molecular features (Fig. 3*D*). It was observed that N104, N665, and N466 were modified with 168, 85, and 59 types of glycosylation, respectively. N508, N197, and N553 were mainly modified with high mannose, whereas N580 were all modified with sialic acid. It indicated the specificity of glycan composition on sites. We also quantified CEA site-specific glycoforms to evaluate the reproducibility of clinical samples (Supplemental Table S10). The Pearson correlation coefficients of three replicates were high with a range of 0.91 to 0.97, which indicated high reproducibility of our method (Fig. 3*E*). However, variations were observed between clinical plasma samples, which showed the heterogeneity between individual patients (Fig. 3*E*). Therefore, our workflow could be used for large-scale analysis of glycosylation of plasma CEA with high reproducibility.

### High-Sensitive CEA Glycosylation Profiling by PRM

**Fig. 4:**
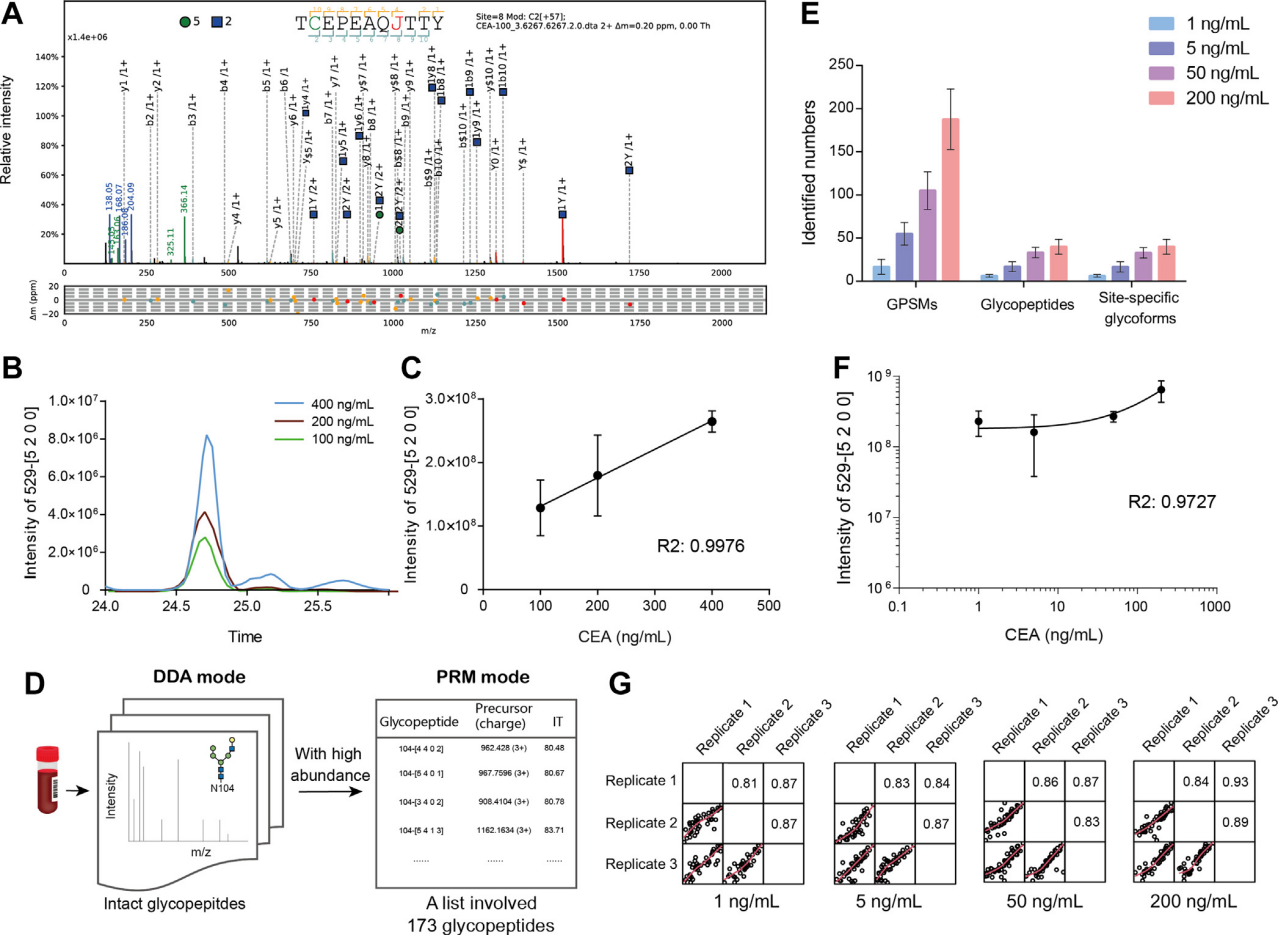
**Limit of detection for CEA glycosylation.** “TCEPEAQJ(N)TTY” with high mannose Hex(5)HexNAc(2) was identified in DDA mode. Its MS/MS spectral annotation was shown when plasma CEA was 100 ng/ml (*A*). Its chromatographic peak intensities (*B*) and linearity (*C*) showed the ability for quantification. Glycosylation of plasma CEA was then profiled in PRM mode. A list of 173 glycopeptides that have high abundance in plasma CEA identification with DDA mode was generated (*D*). Their identification was shown when plasma was 1, 5, 50, and 200 ng/ml (*E*). N529 modified with Hex(5)HexNAc(2) could also be quantified, and R square of its linearity was 0.9727 (*F*). Pearson correlation coefficients for binary comparison of three replicates were calculated when plasma CEA were 1, 5, 50, and 200 ng/ml. Their values were all above 0.8, which indicated the reproducibility of our workflow with PRM mode (*G*). CEA, carcinoembryonic antigen; DDA, data-dependent acquisition; PRM, parallel reaction monitoring.

Based on the comprehensive discovery of plasma CEA glycosylation, we further evaluated the quantification performance of the workflow, which is critical for clinical cohort study with high sensitivity and quantification precision. Patient plasma was diluted according to its CEA concentration of 400, 200, and 100 ng/ml, and its glycosylation was profiled in DDA mode first. N529 from the peptide “TCEPEAQNTTY” modified with high mannose Hex(5)HexNAc(2) was identified with excellent MS/MS match when plasma CEA was 100 ng/ml (Fig. 4*A*), and its intensities from chromatographic peaks increased because of the increase in plasma CEA (Fig. 4*B*). In addition, it showed good linearity with R square 0.9976 according to the quantification *via* pGlycoQuant (Fig. 4*C*). However, only N529 modified with Hex(5)HexNAc(2) and N650 modified with Hex(4)HexNAc(3)NeuAc(1)Fuc(1) could be identified when plasma CEA concentration decreased to 100 ng/ml.

To further increase quantification performance and sensitivity, we went on to contract PRM assay for 173 glycosylated peptides (four are in different charges). These intact glycopeptides were extracted from aforementioned plasma CEA glycosylation identification, which have high abundance in DDA mode (Fig. 4*D*, Supplemental Table S2). The number of GPSMs, intact glycopeptides, and site-specific glycoforms reduced as the plasma CEA levels decreased (Fig. 4*E*, Supplemental Table S11). Thirteen intact glycopeptides could be identified with good precursor peaks and MS/MS matches when plasma CEA was 1 ng/ml (Supplemental Fig. S3), and N529 modified with Hex(5)HexNAc(2) was among them. Its quantification showed good linearity with R square 0.9727 when plasma was 200, 50, 5, and 1 ng/ml (Fig. 4*F*). In addition, Pearson correlation coefficients of replicates in different CEA concentrations were above 0.8, which indicated the reproducibility of the workflow with PRM mode (Fig. 4*G*). Therefore, our chemical proteomic strategy combined with MS analysis in PRM mode could be used for glycosylation profiling of plasma CEA with low concentration.

### Site-Specific Glycoforms of CEA Discriminated Patients With CRC and Lung Cancer

**Fig. 5:**
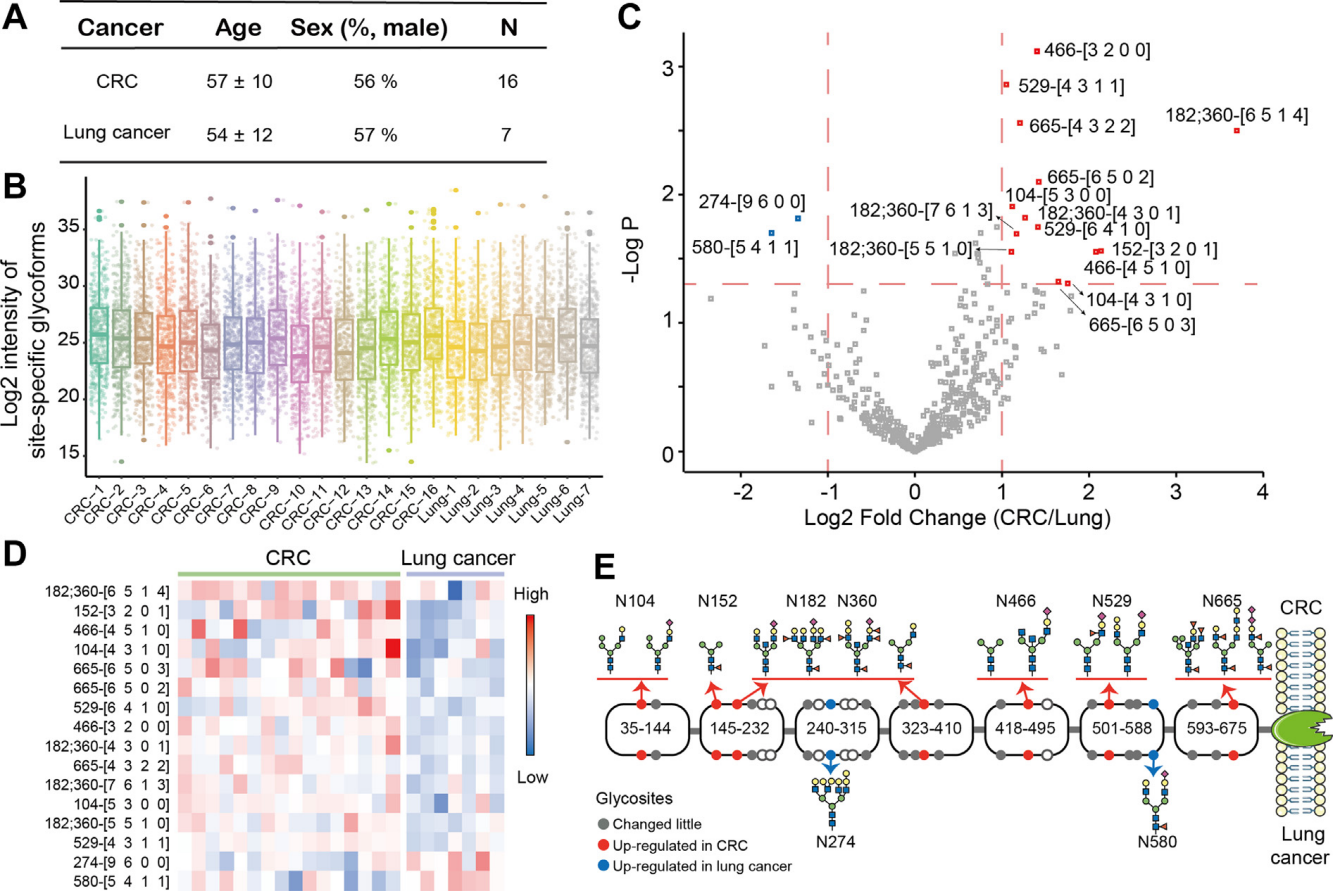
**Site-specific glycoforms of plasma CEA in patients with CRC and lung cancer.** Patients with 16 advanced CRC and seven advanced lung cancer were involved (*A*). Distribution of log_2_-transformed intensities of site-specific glycoforms quantified by pGlycoQuant in 23 patients, and it showed similar distribution (*B*). According to the criteria that the fold change was >2 or <0.5 and *t* test showed significance (*p* < 0.05), 16 site-specific glycoforms were observed to be significantly differential from volcano plot. *Red* and *blue* refers to upregulation in CRC and lung cancer, respectively (*C*). Their expressions in different samples were shown in the heatmap (*D*). The differential sites and plausible glycoforms of CEA were performed. The *upper* and the *lower* showed the upregulation in patients with CRC and lung cancer, respectively (*E*). CEA, carcinoembryonic antigen; CRC, colorectal cancer.

To explore the roles of CEA glycosylation in cancer patients, we investigated their performance in 16 advanced CRC and seven advanced lung cancer patients with CEA overexpressed (>300 ng/ml, Supplemental Table S1), whose age and sex were almost the same (Fig. 5*A*). Intact glycopeptides were detected by LC–MS with DDA mode. Site-specific glycoforms were quantified by pGlycoQuant. Their intensities in 23 patients were drawn and showed similar distribution (Fig. 5*B*, Supplemental Table S12). Based on the quantified site-specific glycoforms, CRC and lung cancer patients could be completely distinguished *via* OPLS-DA model (Supplemental Fig. S4). According to the criteria that the fold change was >2 or <0.5 and *t* test showed significance (*p* < 0.05), 16 site-specific glycoforms were observed to be significantly differential from volcano plot (Fig. 5*C*), and their detail intensities were shown (Supplemental Fig. S5). The heatmap described the expressions of significantly differential site-specific glycoforms in the samples, and 14 and two site-specific glycoforms were upregulated in CRC and lung cancer patients, respectively (Fig. 5*D*). We depicted the changed sites and plausible glycoforms of CEA (Fig. 5*E*). Twenty-two glycosylation sites of CEA were identified, nine of which (N104, N152, N182, N274, N360, N466, N529, N580, and N665) exhibited great change in different cancers. The glycosylation of N274 and N580 was upregulated in patients with lung cancer, whereas others were upregulated in patients with CRC. Therefore, glycosylation features were correlation with the type of cancers, which might contribute to disease diagnosis.

### CEA Site-Specific Glycoforms Changed in CRC Patients in Different Stages

**Fig. 6:**
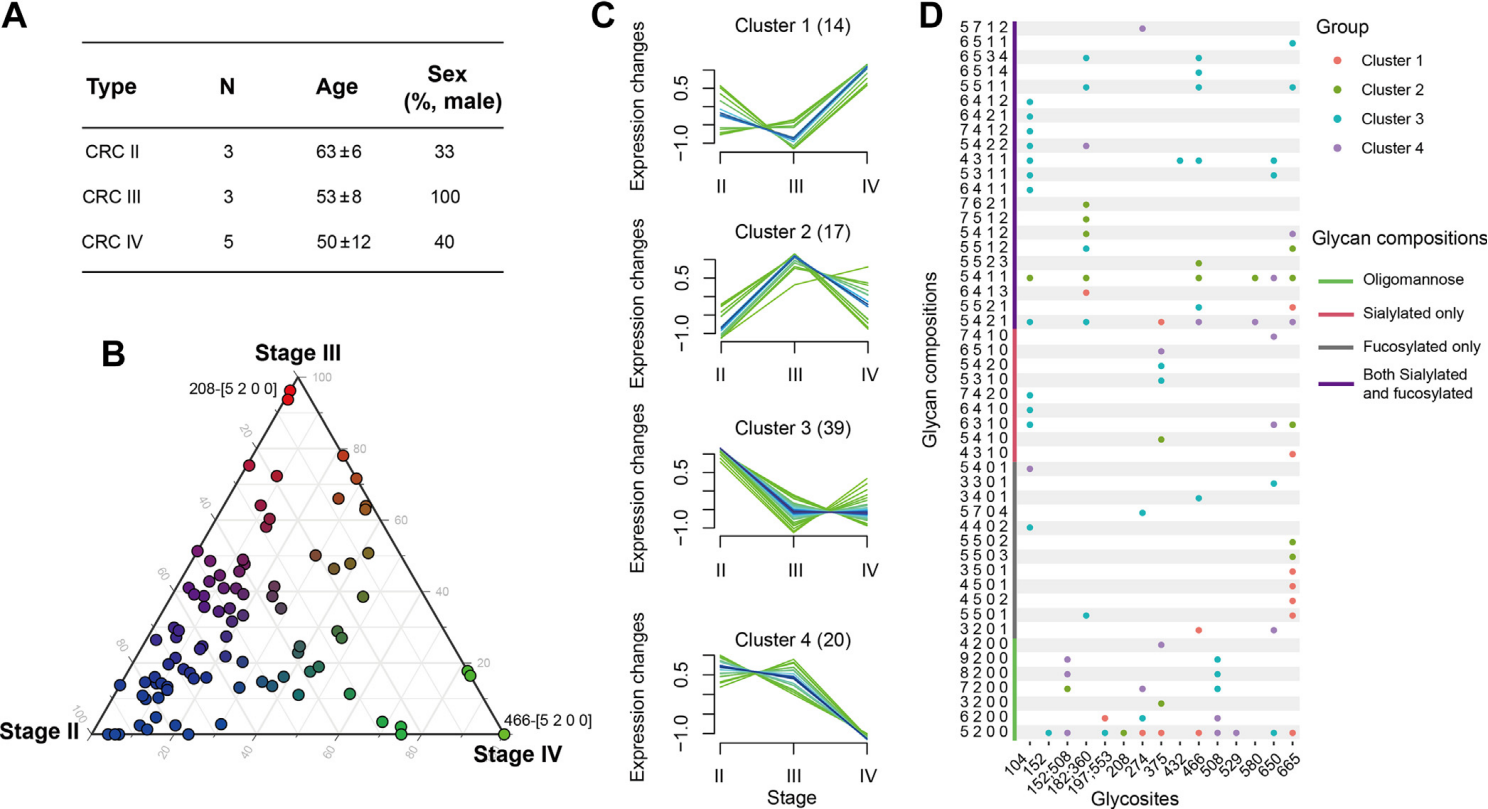
**Site-specific glycoforms of plasma CEA in CRC cancer in different stages.** CRC patients with different stages were involved, which contained three in stage II, three in stage III, and five in stage IV (*A*). The distribution of site-specific glycoforms of CEA in their plasma was shown (*B*). Ninety site-specific glycoforms were divided into four clusters, and 14, 17, 39, and 20 site-specific glycoforms were respectively enriched in cluster 1, 2, 3, and 4 (*C*). The cluster attribution of site-specific glycoforms was shown. The color of *dots* represented different clusters (*D*). CEA, carcinoembryonic antigen; CRC, colorectal cancer.

CEA lacks sensitivity for the diagnosis of CRC, especially in the early stage in which CEA may not be upregulated. We then investigated the CEA glycosylation of CRC patients with different progression, which contained three in stage II (CEA below 2 ng/ml), three in stage III (two with CEA around 3 ng/ml and one with CEA 300 ng/ml), and five in stage IV (CEA above 250 ng/ml) (Fig. 6*A*, Supplemental Table S1). We applied LC–MS/MS with PRM mode for its intact glycopeptide analysis. Because of the individual differences, 90 of 173 site-specific glycoforms were identified and quantified (Supplemental Table S13). Different stages of CRC patients were clustered *via* OPLS-DA model based on quantified site-specific glycoforms (Supplemental Fig. S6). Also, site-specific glycoforms have differential distribution at different stages (Fig. 6*B*). Although CEA was highly overexpressed in stage IV, most site-specific glycoforms were highly upregulated in stage II of CRC. It indicated that CEA glycosylation was additional dimension of molecular features to describe the status of diseases in addition to CEA protein level, and we directly used the intensities of CEA glycosylation for analysis. High-mannose glycan on site N466 was expressed only in stage IV, whereas on site N208 was not in stage IV. Site-specific glycoforms showed different performances as the progression of CRC, and they were divided into four clusters (Fig. 6*C*). Cluster 1 and 2 showed increased trends and dramatically rose at stage IV and III, respectively. Cluster 3 and 4 showed decreased trends and greatly dropped at stage III and IV, respectively. It was observed that 14, 17, 39, and 20 site-specific glycoforms were respectively enriched in cluster 1, 2, 3, and 4 (Supplemental Table S14). Detail information of intact glycopeptides in four clusters were analyzed (Fig. 6*D*). Most glycosylations of N665 tended to increase in stage III or IV as shown in cluster 1 and 2, whereas the glycosylation of N650 exhibited decrease in cluster 3 and 4. Most N508 modified with high mannose were enriched in cluster 3 and 4, and N104 modified with sialic acid and fucose were enriched in cluster 3. Most sialylated or fucosylated glycopeptides were enriched in cluster 3, which indicated that the sialylation or fucosylation was upregulated at stage II and greatly declined at stage III. In conclusion, the features of CEA site-specific glycoforms would change as the state of disease, which would distinguish patients and showed the potential for disease diagnosis.

## Discussion

In this study, we established a chemical proteomic strategy for glycosylation identification of plasma CEA with high sensitivity. A trifunctional probe has been used to generate a covalent link between anti-CEA antibody and CEA, and the strong interaction contributed to the improvement for glycosylation identification of plasma CEA. As much as 26 of 28 N-link glycosylation sites could be identified, and glycosylation of plasma CEA as low as 1 ng/ml could be profiled. The strategy had been applied to 42 clinical samples in this study, and the quantification of site-specific glycoforms exhibited high reproducibility, which indicates that it is able to be used for large-scale clinical sample analysis. Our chemical proteomic strategy could be used for glycosylation identification of other biomarkers with low abundance in complex plasma samples.

Multienzymatic strategy greatly contributed to in-depth glycosylation identification of plasma CEA. Trypsin combined with chymotrypsin has identified 26 of 28 N-linked glycosylation sites when false discovery rate was set below 1%, and it showed the most comprehensive N-glycosylation site characterization of CEA on intact glycopeptide level as far as we known. N288 and N292 could not be identified since their MS/MS spectrum was not well matched. They could not either be identified in trypsin, Glu-C, or pronase digestion (24). This may be due to the complexity of glycopeptide-carried several potential N-glycosylation sites or their absence in the real clinical samples. In addition, some enzymatic glycopeptides would represent two glycosites. Glycopeptide “NVTR” may refer to N204 or N560 because of the multiple cleavage sites, whereas glycopeptides “SNGNRTLTLF” and “WVNNQSLPVSPRLQL” may be N197 or N553 and N182 or N360, respectively, because of the repetitive regions of CEA. N553 and N560 respectively from glycopeptides “SNGNRTLTLFJVTRNDARAY” and “NVTRNDARAY” could be identified, but it was difficult for us to distinguish N182 and N360.

Sialylation and fucosylation were important features of CEA, and they had been shown in eight individuals who were diagnosed with advanced cancer with metastasis. Sialylation is a biologically important modification, and its alternation during cancer progression has been approved as a potential biomarker (28). Compared with commercial CEA, elevated sialylation of CEA was observed in patients with metastasis. It was consistent with the fact that increased sialylation contributed to metastasis (29). Fucosylation has been involved in the tumor recurrence (30), and core fucosylation contributed to dismal prognoses and metastatic potential (31). Fucosylation of CEA from eight individuals was an impressive feature, which accounted for around 70%. Of them, core fucosylation occupied up to 80%. Terminal fucosylation is involved in the formation of Lewis antigens. CEA carrying sialyl-Lewis x is associated with aggressive tumor features and could be a prognosis biomarker (32).

Although CEA has been widely used for cancer diagnosis and prognosis, its glycosylation was overlooked. Our strategy systematically characterized the glycosylation of plasma CEA in cancer patients. The distributions of site-specific glycoforms of plasma CEA differed in patients with CRC and lung cancer as well as in the progression of CRC patients. Most site-specific glycoforms showed upregulation in CRC patients with stage II, which was consistent with the report illustrating the CEA glycosylation from tumor tissues peaked at stage II during CRC progression (22). Thus, N-glycosylation site characterization of CEA on intact glycopeptide level could be used for evaluation of disease, which could provide additional dimension of features to improve the sensitivity and specificity of cancer diagnosis.

## Conclusions

In conclusion, we develop a chemical proteomic method with high sensitivity and reproducibility to comprehensively explore the glycosylation of plasma CEA based on trifunctional probe and multienzymatic approach. Glycosylation of as low as 1 ng/ml CEA could be identified in targeted proteomic analysis mode. This strategy was applied to cancer patients with different stages of CRC and lung cancer. The reliable intact glycopeptide profiling from these clinical samples well support the potential of applying glycosylation features for differentiating cancer patients. Our chemical proteomic strategy therefore provided a generally applicable chemical proteomic method for glycosylation profiling of well-characterized plasma glycoprotein biomarkers.

## Data Availability

Datasets in this study are available at iProx (www.iprox.cn/) with the dataset identifier PXD046518.

## Supplemental data

This article contains supplemental data.

## Conflict of interest

The authors declare no competing interests.
