# Supplementary material for: Chemical Proteomic Approach for In-Depth Glycosylation Profiling of Plasma Carcinoembryonic Antigen in Cancer Patients
Source: Mol Cell Proteomics. 2023 Oct 10;22(11):100662. doi: 10.1016/j.mcpro.2023.100662 (PMC10652130; doi:10.1016/j.mcpro.2023.100662)
Supplement: Supplemental Figures S1–S6 and Tables S1–S14 [file mmc1.docx]

**Supporting Information**

**Chemical proteomic approach for in-depth glycosylation profiling of plasma carcinoembryonic antigen in cancer patients**

Jin Chen^1, 2, #^, Lijun Yang^3, #^, Chang Li^1^, Luobin Zhang^3^, Weina Gao^1^, Ruilian Xu^3, *^, Ruijun Tian^1, *^

^1^Department of Chemistry and Research Center for Chemical Biology and Omics Analysis, School of Science, Southern University of Science and Technology, Shenzhen 518055, China.

^2^Clinical Center for Molecular Diagnosis and Therapy, the Second Affiliated Hospital of Fujian Medical University, Quanzhou, Fujian, 362000, China.

^3^Department of Oncology, The Second Clinical Medical College, Jinan University (Shenzhen People's Hospital), Shenzhen 518020, China, The First Affiliated Hospital, Jinan University, Guangzhou 510632, China

^#^Jin Chen and Lijun Yang contributed equally to this work.

*Corresponding author:

Ruilian Xu, [xuruilian@126.com](mailto:xuruilian@126.com)

Ruijun Tian, [tianrj@sustech.edu.cn](mailto:tianrj@sustech.edu.cn)

**Contents**

Figure S1. Compare to without UV irradiation, much less peptides from non-specific proteins were observed (A) and GPSMs of CEA accounted for a two-fold higher proportion of all GPSMs (B) with UV.

Figure S2. The expressions of commercial and plasma CEA were quantified according to its non-glycosylated CEA peptides.

Figure S3. The precursor peaks and MS/MS matches of thirteen intact glycopeptides when plasma CEA was 1 ng/mL.

Figure S4. The plasma samples from patients with CRC and lung cancer were classified according to their CEA site-specific glycoforms via OPLS-DA

Figure S5. The intensity of significantly differential site-specific glycoforms of plasma CEA in patients with CRC and lung cancer.

Figure S6. The plasma samples from CRC patients in different stages were classified according to their CEA site-specific glycoforms via OPLS-DA.

Table S1. Detail information of clinical samples.

Table S2. PRM list of CEA intact glycopeptides.

Table S3. Glycosylation identification of plasma CEA based on chemical labeling (with and without UV) under three replicates.

Table S4. Glycosylation identification of plasma CEA with different concentration of trifunctional probe under three replicates.

Table S5. Glycosylation identification of plasma CEA with different amount of antibody under three replicates.

Table S6. Glycosylation identification of commercial CEA using trypsin paired with either chymotrypsin or elastase.

Table S7. Glycosylation identification of commercial CEA in plasma with multi-enzymatic digestion under three replicates.

Table S8. Quantification of site-specific glycoforms of CEA in plasma digested by multienzymes.

Table S9. Glycosylation identification of plasma CEA in 8 individuals with three replicates.

Table S10. Quantification of CEA site-specific glycoforms in 8 individuals.

Table S11. Intact glycopeptides identified by MS with PRM mode.

Table S12. Quantification of CEA site-specific glycoforms in patients with CRC and lung cancer.

Table S13. Quantification of CEA site-specific glycoforms in CRC patients at different stage.

Table S14. The CEA site-specific glycoforms in different clusters.



Figure S1 Compare to without UV irradiation, much less peptides from non-specific proteins were observed (A) and GPSMs of CEA accounted for a two-fold higher proportion of all GPSMs (B) with UV.



Figure S2. The expressions of commercial and plasma CEA were quantified according to its non-glycosylated CEA peptides. According to the search results, the logarithms of commercial and plasma CEA intensities were 23.4 ± 0.6 and 23.4 ± 0.2, respectively. That is, their expressions were roughly equal.


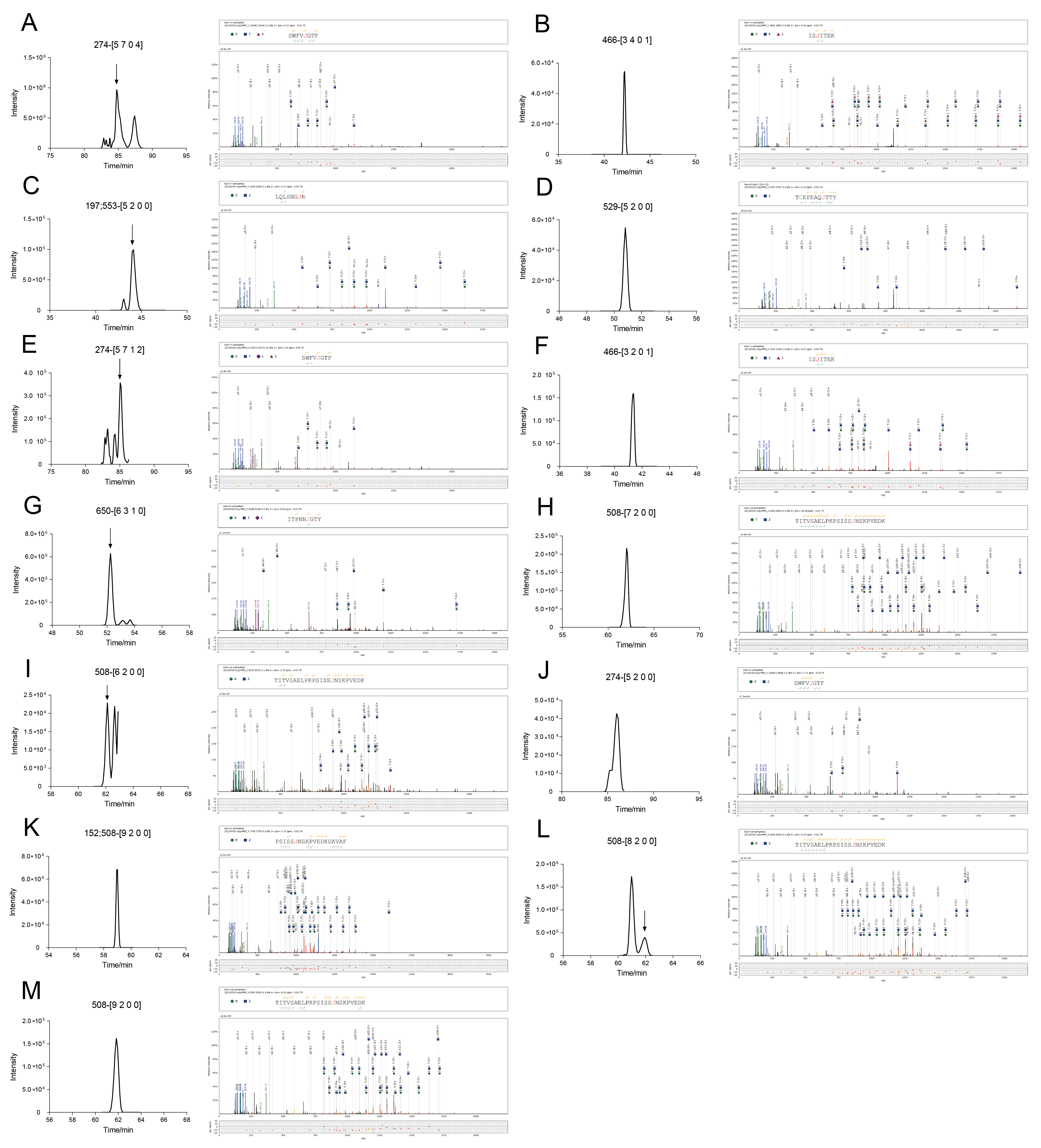
Figure S3. The precursor peaks and MS/MS matches of thirteen intact glycopeptides when plasma CEA was 1 ng/mL.


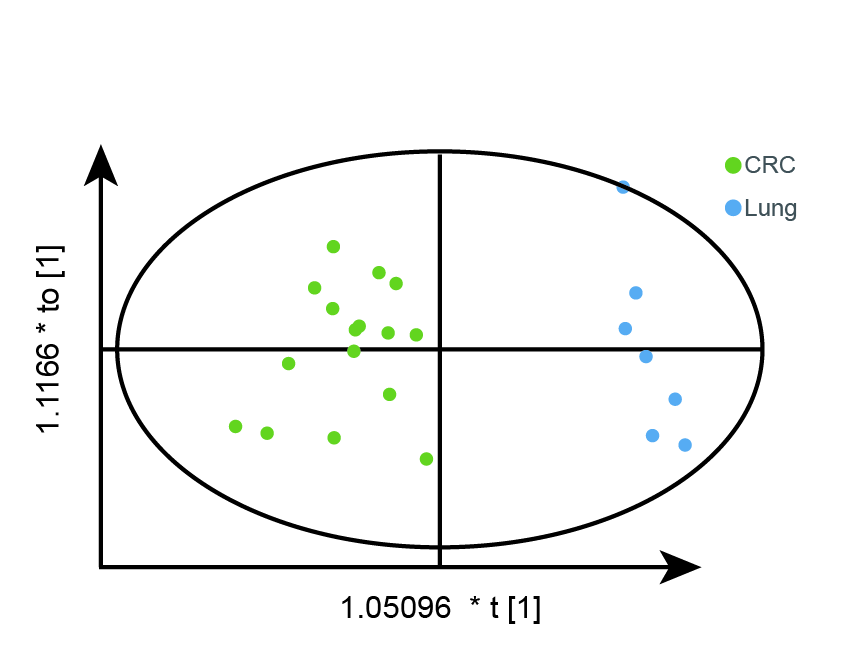


Figure S4. The plasma samples from patients with CRC and lung cancer were classified according to their CEA site-specific glycoforms via OPLS-DA.





Figure S5 The intensity of significantly differential site-specific glycoforms of plasma CEA in patients with CRC and lung cancer.


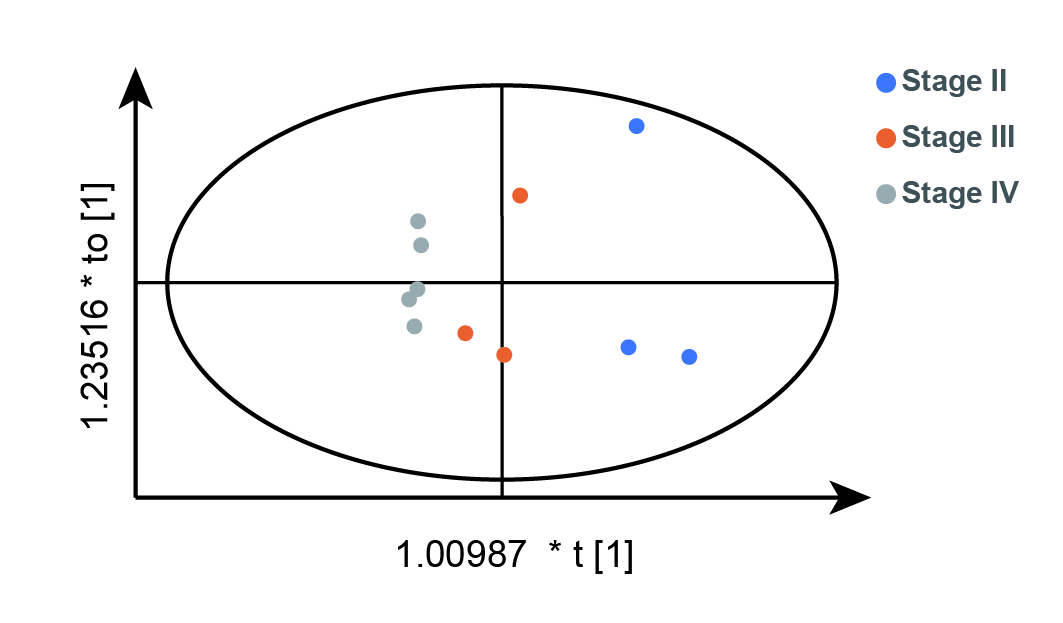
Figure S6. The plasma samples from CRC patients in different stages were classified according to their CEA site-specific glycoforms via OPLS-DA.
